# NKX3.1 Expression Contributes to Epithelial–Mesenchymal Transition of Prostate Cancer Cells

**DOI:** 10.1021/acsomega.3c03127

**Published:** 2023-09-01

**Authors:** Iroda Saydullaeva, Bilge Debelec Butuner, Kemal Sami Korkmaz

**Affiliations:** † Faculty of Engineering, Department of Bioengineering, Cancer Biology Laboratory, Ege University, Izmir 35040, Turkey; ‡ Faculty of Pharmacy, Department of Pharmaceutical Biotechnology, Ege University, Izmir 35040, Turkey

## Abstract

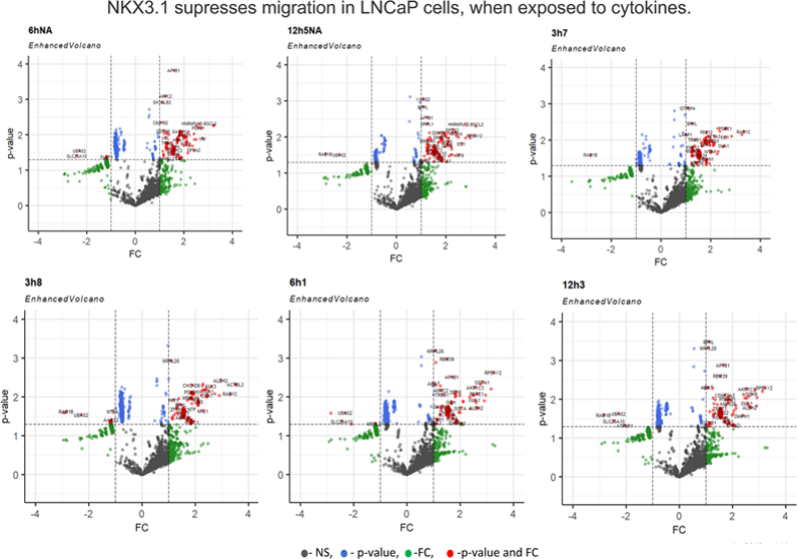

Studies demonstrate that inflammation synergizes with high-grade aggressive prostate tumor development and ultimately metastatic spread, in which a lot of work has been done in recent years. However, the clear mechanism of inflammation inciting prostate cancer remains largely uncharacterized. Our previous study has shown that the conditioned media (CM)-mediated LNCaP cell migration is partially correlated with the loss of expression of the tumor suppressor NKX3.1. Here, we continue to investigate the inflammation-mediated migration of prostate cancer cells, and the role of NKX3.1 in this process to gain insights into cell migration-related changes comprehensively. Earlier, the model of inflammation in the tumor microenvironment have been optimized by our research group; here, we continue to investigate the time-dependent effect of CM exposure together with NKX3.1 changes, in which we observed that these changes play important roles in gaining heterogeneous epithelial-to-mesenchymal transition (EMT) phenotype. Hence, this is an important parameter of tumor progression; we depleted NKX3.1 expression using the CRISPR/Cas9 system and examined the migrating cell clusters after exposure to inflammatory cytokines. We found that the migrated cells clearly demonstrate reversible loss of E-cadherin expression, which is consistent with subsequent vimentin expression alterations in comparison to control cells. Moreover, the data suggest that the AR-mediated transcriptional program also contributes to mesenchymal-to-epithelial transition (MET) in prostate cancer progression. Furthermore, the quantitative proteomic analysis showed that migrated subpopulations from the same cell line presented different phenotypes in which the proteins overexpressed are involved in cell metabolism and RNA processing. According to KEGG pathway analysis, the ABC transporters were found to be the most significant. Thus, the dynamic process of cellular migration favors diverse genetic compositions under changing tumor microenvironments. The different levels of invasiveness are supported by shifting the cells in between these EMT and MET phenotypes.

## Introduction

Prostate cancer (PCa) is considered as a chronic disease and the second most frequent malignancy in men worldwide.^1^ The incidence and mortality of prostate cancer correlate with an increasing average age of 66 years at the time of diagnosis. Of note, for African-American men, the incidence rates are higher than for white men with 158.3 new cases diagnosed per 100,000 and the mortality rate is approximately twice.^2^ Though PCa is a severe disease, most diagnosed cases of PCa do not result in fatality in a lifetime. The 5-year relative survival rate of PCa diagnosed at a local stage approaches 100%.^3^ Unfortunately, once PCa has spread to the lymph nodes and bones, the outcome is commonly poor with a relatively low survival rate, which is one-third of that for organ-confined disease.^4^ Statistically, 25% of men with PCa worldwide develop metastatic disease, and the 5-year survival of patients with metastasis to a distant site is significantly reduced to 29%.^1,5^

Epidemiological studies show that PCa development is hormone-dependent at the initial stages and is strongly associated with inflammation. Putative susceptibility genes involved are *RNAseL, MSR1*, *TLR4, MIC1, PON1, BRCA2, CHEK2, NKX3.1*, and *OGG1*. Since these genes have important roles in diverse cell types and carcinogenesis processes, their functional roles have also been considered in infection, inflammation, and oxidative stress response pathways.^6,7^ However, a significant correlation between suppression of PCa incidence and progression with inhibition of prostate inflammation has not been clearly shown yet.^8^

Inflammatory responses may play decisive roles at different stages of tumor development, involving initiation, progression, malignant transformation, invasion, and metastasis.^9^ During the multistage carcinogenesis, depending on the tumor microenvironment, and also the genetic makeup, cells may acquire migration potential, which is fundamental for aggressive metastatic disease progression. This is achieved by reactivating a latent embryonic program: epithelial-to-mesenchymal transition.^10^ In the epithelial-to-mesenchymal transition (EMT) process, epithelial cells fail to maintain apical–basolateral polarity and cell-to-cell adhesion because of an altered transcriptional program, which eventually may lead to mesenchymal phenotype. The key epithelial marker localized to adherent junctions is E-cadherin, which enables the cells to maintain their epithelial phenotypes. Nevertheless, the EMT event might be induced by E-cadherin loss that can be carried out by certain transcription factors, including Snail Family Transcriptional Repressor 1 (Snail), Snail Family Transcriptional Repressor 2 (Slug), Zinc finger E-box binding homeobox (ZEB1), ZEB2, Kruppel-like factor 8 (KLF8), and Twist-related protein 1 (Twist1). In addition to E-cadherin loss, increased N-cadherin, Fibronectin, and Vimentin expressions are also seen as consequent to cellular migration, indicating EMT activation. It has been recently suggested that EMT is a reversible process, and is recognized as a bidirectional switch in the latent embryonic program as well as in carcinogenesis that is maintained by several signaling pathways such as Transforming Growth Factor β (TGF-β), Wnt, β-Catenin, Notch, Hedgehog, and receptor tyrosine kinases, depending on cell type and the stages of development.^11,12^

*NKX3.1* is an androgen-regulated gene that affects early events in the response to DNA damage and its expression results in increased cell survival and clonogenicity. The decreased level of NKX3.1 is attenuated to the acute response to DNA damage.^13−15^ Previously, Khalili et al. have shown that the NKX3.1 expression is lost in some of the ducts of the prostate gland in mice, which is correlated with increased T cell and macrophage infiltration.^16^ In addition to this study, we also have developed an approach to investigate the genetic alterations upon exposure to an inflammatory microenvironment^17^ and observed a clear decrease in Androgen Receptor (AR) expression and -mediated gene expression of NKX3.1, consistent with previous studies.^15,18^ In these studies, we intriguingly found that some of the glands still kept the NKX3.1 expression.^19^ As NKX3.1 expression is lost in organ-confined tumors whereas restored in high-grade metastatic tumors of the prostate,^14^ these data led us to develop an *in vitro* approach to simulate the inflammatory microenvironment to investigate the related genetic variations.

We studied the role of NKX3.1 in cell migration by optimizing cytokine exposure to mimic the inflammatory microenvironment in LNCaP cells. Then, we isolated the aggressive migrating clones, indeed originating from LNCaP cells, and named them as subpopulations based on their time of exposure to inflammatory cytokines. The collected medium assigned as “CM” was collected from the PMA-differentiated U937 monocyte cell line upon induction with Lipopolysaccharides (LPS). TNFα, IL6, and IL-1β levels were measured and optimized to increase the experimental reproducibility of the “*in vitro* inflammation model”, which was explained previously.^17^ When the LNCaP cells were treated with different concentrations of CM, the cells exhibited subpopulations with different NKX3.1 expression levels. When we also checked them for the E-cadherin, Vimentin, and N-cadherin expression levels, we observed specific subdivisions having variations in morphology from epithelial to mesenchymal. Hence, most of the other subpopulations represented intermediate forms with varying expressions, which were also studied additionally.

## Materials and Methods

### Cell Culture

LNCaP and U937 cells were obtained from the American Type Culture Collection (ATCC, Manassas, VA). They were propagated using an RPMI 1640 medium supplemented with 10% fetal bovine serum, l-glutamine (2 mM), penicillin (100 U/mL), and streptomycin (100 mg/mL), mixed as discussed previously.^17^ Cells were grown in a humidified incubator containing 5% CO_2_ at 37 °C. Basically, cells with passage numbers 5–15 were used for experiments. As the cells were cultivated, their phenotypes were checked morphologically under a microscope. The contamination of the cell culture with mycoplasma was checked periodically and, if necessary, by staining with DAPI.

### Macrophage Differentiation and Collection of Conditioned Media (CM)

The U937 monocyte cell line was used as a model to induce macrophage function. To accomplish cytokine production upon macrophage differentiation, cells (8 × 10^6^) were seeded into 75 cm^2^ culture flasks for 2 h prior to the treatments. Next, PMA (phorbol 12-myristate 13-acetate) was added at a concentration of 16 nM for 16 h, and the adherent “grape bunch-like” clusters were followed by light microscopy. After the cells were washed with the medium, they were allowed to rest for 2 h and LPS was added at a final concentration of 10 ng/mL. The cells were incubated for 24 h and the supernatant conditioned medium (CM) was collected and filtered using 0.8 and 0.22 μm filters sequentially for further use.

### Measurement of Cytokines in the CM and Labeling the Clones

Before feeding the LNCaP cells with the collected CM, TNFα (Invitrogen, Carlsbad, CA), levels were examined using ELISA according to the manufacturer’s instructions (detection limits are specified as 15.6–1000 pg/mL).^17^ The overall design of our study was to study time-dependent changes in tumor cells in an inflammatory microenvironment. In contrast to previous studies, we continued to grow migratory cells and found that only a few of them continued to survive. We aimed to select a single colony for further study from migratory cell samples after 3, 6, and 12 h of CM application. Each experimental group had a different number of colonies since the application of CM depending on the time had a different effect on the behavior of the migrated cells.

### Treatments

All experiments were carried out using cells grown in a humidified incubator containing 5% CO_2_ at 37 °C. The treatments to induce inflammatory microenvironment were performed with CM (500 pg/mL TNFα) at appropriate periods of 3, 6, and 12 h. TNFα concentrations were adjusted by diluting the CM using an RPMI 1640 medium, described in detail previously.^17^

### Cell Migration Assay

The cell migration assay was carried out using trans-well inserts placed in six-well plates, which had a pore size of 8.0 μm (Corning, NY). To determine migration in LNCaP cells, the cells were seeded in 10 cm plates and allowed to reach 70–80% confluence, followed by incubation with or without CM, including 500 pg/mL TNFα for different time points (3, 6 and 12h). After incubation, approximately 3 × 10^5^ cells in 1.5 mL of an RPMI1640 culture medium without FBS were replaced in the upper compartment of six-well plates, and the lower compartment was filled with 2 mL of RPMI 1640 supplemented with 10% FBS. LNCaP cells were incubated for 72 h at 37 °C in 5% CO_2_. Next, trans-well inserts were removed, and the number of migrated cells to the lower compartment was controlled by light microscopy. Migrated cells were grown for further analysis.

### Subcellular Fractionation

LNCaP cells (2 × 10^6^) were washed with PBS and pelleted for 5 min at 300 g. The cell pellet was resuspended in 500 μl of buffer A (250 mM sucrose, 50 mM Tris–HCl pH: 7.4, 5 mM MgCl_2_), and cell lysis was performed by sonication on ice (3 times 10 s pulse with 40% power and 30 s interval). The suspension was centrifuged at 800 g for 15 min and pellet A was saved to isolate nuclei. Supernatant A was centrifuged again at 1000*g* for 15 min. Supernatant B was saved to isolate the cytosolic proteins. Pellet A, saved for isolation of nuclei, was dissolved in 500 μL of buffer A and centrifuged at 1000*g* for 15 min. Supernatant C was added to supernatant B for isolating cytosolic proteins and stored on ice until then. Pellet C was resuspended in 500 μL of buffer B1 (1 M sucrose, 50 mM Tris–HCl pH, 5 mM MgCl_2_) and layered onto a 1.5 mL cushion of buffer B2 (2 M sucrose, 50 mM Tris–HCl pH: 7.4, 5 mM MgCl_2_). Then, centrifugation at 2100*g* for 1 h was done. Pellet D was taken up in 250 μL of buffer D (20 mM HEPES, pH: 7.9, 1.5 mM MgCl_2_, 0.5 M NaCl, 0.2 mM EDTA, 20% glycerol, 1% Triton X-100) and incubated for 1 h by rotating at 30 rpm. The suspension was sonicated on ice again (3 times 10 s pulses with 40% power and 30 s interval) and centrifuged at 9000*g* for 30 min. The nuclear protein lysate was collected as the supernatant. Furthermore, the pooled supernatants B and C were centrifuged for 150 min at 16,000*g* to isolate the cytosolic protein lysate from the supernatant. The pellet was dissolved in 250 μL of buffer C (20 mM Tris–HCl pH: 7.4, 0.4 M NaCl, 15% glycerol, 1.5% Triton X-100), incubated for 1 h by rotating at 30 rpm, and centrifuged at 9000*g* for 30 min to isolate membrane components from the supernatant. All incubations and centrifugations were carried out at 4 °C and buffers were supplemented with 1× protease inhibitor cocktail (Roche GmbH, Germany) as well as 1 mM DTT directly before use. Then, 50 μg of each fraction was subjected to sodium dodecyl sulfate-polyacrylamide gel electrophoresis (SDS-PAGE) for western blotting, as described in detail previously.^17,19^

### Western Blotting Analysis

Protein concentrations were determined using BCA assay (Sigma, U.K.). SDS-PAGE and western blotting were performed under standard conditions using 50 μg of the protein lysate per lane; proteins were separated on a 10–20% gel and transferred to a PVDF membrane (Amersham, U.K.) using a wet transfer blotter. The PVDF membrane was blocked with 5% dry milk in TBS-T (Tris–borate–saline solution containing 0.1% Tween 20). Primary and secondary antibody incubations were performed using TBS-T containing 0.5% dry milk or 5% BSA at RT for 1 h or at 4 °C overnight. Membranes were developed using an ECL plus reagent (Amersham, U.K.) for 5 min and were photographed using Kodak X-ray films in a dark room, as performed previously.^17,19^

### Immunofluorescence Labeling and Microscopy

Cells were grown on coverslips and CM treatment was performed. At the time of analysis, cells on coverslips were rinsed with PBS, fixed with methanol at −20 °C for 30 min, permeabilized with 0.2% Triton X-100 in PBS for 5 min on a shaker and blocked for 5 min using 1% BSA in PBS. Primary antibodies (in 1% BSA/PBS) were added and incubated in a humidified chamber for 1 h, and cells were washed twice with PBS. Secondary antibody incubations were performed at RT for 20 min using Alexa Fluor 488 (antirabbit) and/or Alexa Fluor 594 (antimouse) antibodies. Finally, cells were washed twice with PBS and mounted on coverslips with 30% glycerol in PBS including 0.5 μg/ml DAPI and analyzed immediately using a Leica DM4000B LED fluorescent microscope (Leica, Germany). Images were captured using Leica imaging software. All labelings were performed as described in detail in our previous studies.^17,19^

### Transfections

To suppress NKX3.1 protein expression, the NKX3.1 CRISPR KO plasmid (Santa Cruz Inc., Germany) was transfected using a Lipofectamine 2000 (Thermo Fisher) transfection reagent. Briefly, LNCaP cells (5 × 10^5^) were seeded into a 35 mm culture plate. When cells achieved 80–90% confluency, the medium was changed to OptiMEM (Gibco) reduced serum medium, and a transfection mix was prepared by adding 1 μg of DNA and 4 μL of Lipofectamine 2000 into 100 μL of OptiMEM medium separately and incubating for 5 min at real-time. After two tubes were mixed and incubated for 15 min, the transfection mix was added to cells dropwise. After 3 h of incubation, the transfection medium was removed and prewarmed RPMI medium including 10% FBS was added. Cells were incubated for an additional 48 h.

To quantify the transfection efficiency of NKX3.1-silenced cells, immunofluorescence images were taken and analyzed by the Fiji platform. The total number of cells was determined by counting DAPI-stained cells, and the number of cells having CRISPR-GFP expression was counted by images in the green channel. NKX3.1 transfection efficiency was quantified by calculating the ratio of the number of GFP-positive cells to the number of total DAPI-positive cells.

### Antibodies

The following antibodies were purchased from manufacturers: E-Cadherin, N-Cadherin, β-Catenin, AR, Cyclin D1, p-H3^(S10)^, Vimentin, BMP-2, BMP-4, MMP-13, DNMT-3α, (Santa Cruz Inc., Germany), p-β-Catenin^(S552)^, β-Actin (Cell Signaling), MMP-9 (Millipore, MA), and SIRT1 (Sigma, MO). The HRP-conjugated antimouse, antirabbit, and antigoat (Amersham, U.K.) and Alexa Flour 488 and 594-conjugated (Invitrogen, CA) secondary antibodies were purchased and used as recommended.

### Reporter Assay

Inflammation-related migratory LNCaP subclones (2.5 × 10^4^) were seeded in a 24-well plate and cultured at 37 °C for 48 h. Then, clones were transfected with pGL3-Basic and pProbasin vectors for 24 h. Then, the cells were treated with 10 nM R1881 for 6 h and then washed with PBS and collected with passive lysis buffer. Luciferase activity was measured by using a dual-luciferase reporter activity kit (Promega, Mannheim, Germany) according to the manufacturer’s protocol.

### Colony Formation Assay

LNCaP subclones (10 × 10^3^ cells/well) were seeded on with or without poly-d-lysine in six-well plates, maintained in a humidified atmosphere comprising 95% air and 5% CO_2_ at 37 °C for 12–14 days. Cell culture media were changed every 3 days. Then, cells were fixed with 4% paraformaldehyde at 25 °C for 20 min and stained using a crystal violet solution (0.05% crystal violet, 1% formaldehyde, 1% methanol and 1× PBS) at 25 °C for 30 min. The stained cells were washed with water by gently dropping and air-dried at room temperature. The numbers of colonies were quantified using the Image J program (version 1.8.0; National Institutes of Health, Bethesda, MD).

### Mass Spectrometric Analysis

The peptides were subjected to a reverse-phase nanoLC-MS/MS (EASY-nLC, Thermo) connected to a Q-Exactive quadrupole Orbitrap mass spectrometer (Thermo Fisher Scientific, Bremen). Samples were directly loaded onto an in-house packed 100 μm i.d. × 17 cm C18 column (Reprosil-Gold C18, 5 μm, 200 Å, Dr. Maisch) and separated at 300 nL/min with 75 or 90 min duration. Linear gradients were applied increasing from 5 to 40 or 30% acetonitrile in 0.1% formic acid, respectively. Survey spectra were acquired on the Orbitrap with a resolution of 70,000, a mass range of 350–1500 *m*/*z*, automatic gain control (AGC) target of 1e^6^, and a maximum injection time of 250 ms in positive mode. Ten most intense ions were selected and then fragmented for analysis in the Orbitrap. MS2 analysis consisted of collision-induced dissociation (higher-energy collisional dissociation (HCD)) (resolution 17 500; AGC 1e6; normalized collision energy (NCE) 26; maximum injection time 85 ms). The isolation window for MS/MS was 2.0 *m*/*z*.

### Data Processing

All raw data files were processed and quantified with Proteome Discoverer (PD) (version 1.4, Thermo Scientific). The search was performed against the Homo sapiens subset of the Uniprot database using Mascot (version 2.5.1, Matrix Science, London, U.K.). PD search settings were as follows: the enzyme was itemized as trypsin and the fragment ion type was electrospray ionization quadrupole time-of-flight. A mass tolerance of 20 ppm for precursor masses and 0.05 Da for fragment ions were used, two missed cleavages were allowed, and cysteine carbamidomethylation was set as a fixed modification. Each peptide spectral match (PSM) (Mascot peptide score ≥25) was isolated from the data in PD. A threshold of at least 0.70 probability was used. The 2plex dimethyl-based quantitation method was chosen in PD, with a mass precision requirement of 2 ppm for consecutive precursor mass measurements. The decoy-based strategy was used to control the peptide and protein false discovery rate (FDR). The search parameters were the following. The filter in PD was set to select only the peptides with “high confidence.” A retention time tolerance of 0.2 min for isotope pattern multiplets was applied and the allowed spectra with a maximum of one missing channel with the integrated Percolator-based filter using a false discovery rate of 1% was quantitated (the results are presented as Supporting Data).

### Statistical Analysis of Proteomics Data

The peptide spectrum match (PSM) score of MS analysis was used for further statistical analysis. The quality of technical replicates was analyzed by using a scatter plot, which displays the relationship between two variables *x* and *y*. The package “ggpubr” was used for this in R software version 4.1.3. Since each selected subpopulation remains unique according to the CM treatment time point and the NKX3.1 expression status, further statistical analyses were performed separately for each subpopulation compared to the control group. Data transformation, normalization, and protein fold change and *p*-value calculation were performed according to guideline Sidoli^20^ by using the Microsoft Excel plug-in Real Statistics (http://www.real-statistics.com). Briefly, proteomics data sets of control and one of the subpopulations were used. For the relative quantification of proteins between the two conditions, proteins that do not have quantitative values have been removed and valid values counted. Next, data were transformed by “log 2” logarithm transformation. Normalizing the abundances across all of the proteins from each sample has been done in two steps: scaling each value against the average and normalization by the slope. Next, the imputation of missing values was replaced by low abundance resampling, where low abundance was intended as ≥2 standard deviations smaller than the distribution of all valid values, and the resampling variability was set to 0.3 (reasonable variability in proteomics experiments). A two-tailed paired t-test was used to calculate the *p*-value, and the *p* ≤ 0.05 threshold value was applied in the selection of proteins for further bioinformatic analysis.

Further bioinformatic analyses of proteomic data were carried out in the R studio platform. Visualization of the direction, magnitude, and significance of changes in gene expression was performed by the volcano plot. For the volcano plot design, the “Enhanced Volcano” package was used.^21^*p*-values below 0.05 were selected for further analysis. The relationship between subpopulations was implemented by the Venn diagram, which was performed by the “gg Venn Diagram” package.^22^ The multiscatter plot and the Pearson correlation score were implemented by the “psych” package to analyze the difference between sample types. The hierarchical heatmap was created under “Complex Heatmap”, providing comprehensive heatmap visualization functionalities.^23^ Functional enrichment analysis was made by the clusterProfiler package.^24^

### Data and Statistics

Statistical analysis of in vitro experiments was performed using Prism software (v9.0, GraphPad Software Inc.) and MS Excel. All experiments were repeated at least three times and the data were expressed as a mean value +/– standard error. The differences in the mean values between the groups were analyzed by a two-tailed Student’s t-test and significant values were represented.

## Results

### Characterization of Inflammation-Mediated Migration in LNCaP Cells

LNCaP cells are androgen-sensitive adherent epithelial cells that grow in aggregates as well as in single cells, and they express NKX3.1 at higher levels upon androgen induction.^18^ In our previous study,^17^ an inflammatory microenvironment model has been established to mimic inflammation-induced cellular alterations in prostate tumorigenesis. LNCaP cells with varying time points of CM treatment exhibited unique cellular morphology and characteristics as well as the migration rate.^19^ In this study, we also used the model to isolate and characterize migrated LNCaP cells upon CM treatment as well as to investigate the inflammatory microenvironment-mediated prostate cancer migration. To collect migrated cells after CM treatment, cells were kept in Boyden chambers for 72 h, washed with a medium, and propagated at normal culture conditions. When the cells were grown enough, they were used for further characterizations. The rate of migration in these cells was found to be reproducible and consistent with the previous data.^19^

**Figure 1:**
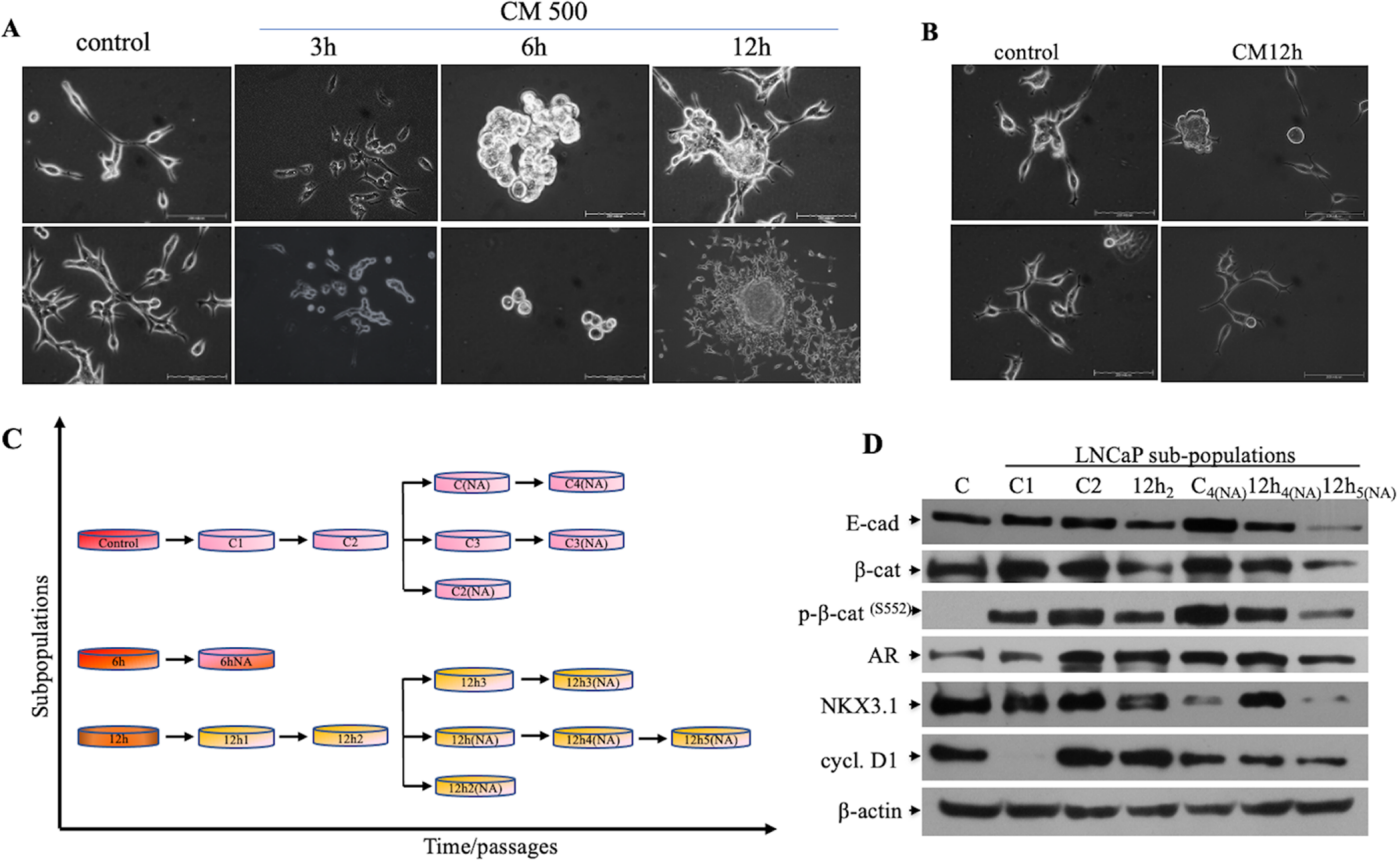
Migrated cells display heterogeneity in a model of the inflammatory microenvironment. (A) Representative light microscopy images of migrated LNCaP subpopulations; subpopulations varied depending on the duration of CM treatment (scale bar: 200 μm). (B) Cells undergoing the suspension cell stage, named nonadhesive (NA). (C) Hierarchical scheme of the obtained subpopulations; NA refers to the group of cells that has passed through the nonadhesive stage. (D) Investigation of proteins involved in cell migration and proliferation by western blotting analysis in selected subpopulations.

LNCaP cell morphology changed upon CM exposure and altered over time (3, 6, and 12h). Migrated cells showing various adhesion and proliferation properties and different morphology at each time point were cultured as subclusters (Figure 1A). For instance, the number of migrated cells was higher in the 3 h CM-treated cell group, but these cells did not keep their viability and were lost at prolonged incubation. Therefore, initial experiments were conducted with the 6 and 12 h of CM treatment groups beside the untreated control cells. The migrated cells showing adhesive and nonadhesive properties were also cultured as separated clusters. In the period of incubation, some of the migrated cells from the control and the 12 h CM-treated group represented nonadhesive morphology and continued to grow as suspension clusters; so, these cells were isolated and grown as separate cultures (Figure 1B). Distinct from the 12 h CM-treated group, cells exposed to 6 h of CM had different characteristics, having low proliferation, and the clump of free-floating cells suspended in a medium survived in the culture for up to 3 months but did not proliferate. We obtained only one subpopulation from these cells. All subpopulations of migrated groups were summarized in the hierarchical scheme (Figure 1C). Since 3 h of CM treatment resulted in the loss of viability, we suggested that these cells shifted to an anchorage-independent stage as a first response of a short period exposure to cytokines, which needs more studies to conclude.

For a detailed investigation of EMT-related protein alterations in migrated cells, E-cadherin, β-catenin, phospho-β-catenin^(S552)^, AR, transcriptionally androgen-regulated NKX3.1, and cyclin D1 protein levels were analyzed in western blotting. As shown in Figure 1D, the epithelial cell marker E-cadherin and β-catenin expressions decreased in 12 h CM-treated migrating subpopulations in comparison to controls. Consistent with β-catenin changes, phospho-β-catenin^(S552)^ increased in all migrated cells, suggesting that the Akt kinase-mediated phosphorylation might lead to the increased survival of the cells. AR expression increased in migrated cells, but intriguingly, native NKX3.1 expression decreased especially in the anchorage-independent growing 12h_5(NA)_ subpopulation, consistent with previous studies. As we observed, all chosen subpopulations displayed distinct expression characteristics, and the anchorage-independent growing cells showed decreased levels of cyclin D1 expression, which might indicate that the cells have a shorter G1 phase than their original counterparts (Figure 1D).

### Time of Exposure to an Inflammatory Microenvironment Determines the Degree of EMT

**Figure 2:**
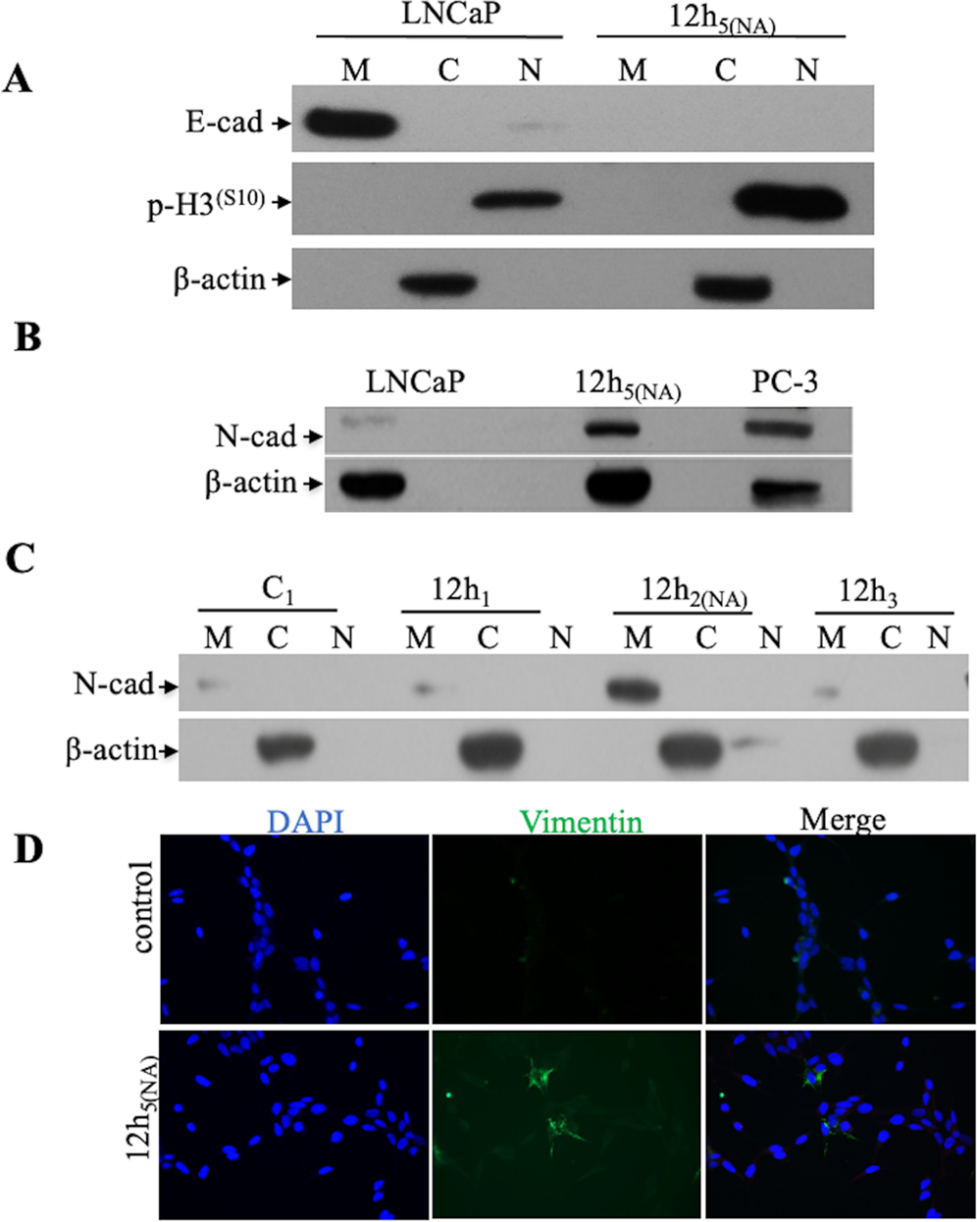
Specific invasive phenotype of chosen cells represented by EMT-related markers. (A) Loss of E-cadherin and overexpression of proliferative p-H3^(S10)^ in 12h_5(NA)_ cells. Primary LNCaP cells were chosen as a control. (B) 12h_5(NA)_ cells simultaneously gained N-cadherin expression. Metastatic PC-3 cells were used as a positive control. (C) Other subpopulations from 12 h CM-treated and migrated cells together with migrated control cells showed either enhanced or decreased expressions of N-cadherin, representing the heterogeneity of migrated cells. (D) Immunofluorescence images of 12h_5(NA)_ and 6h_(NA)_ subpopulations stained with the mesenchymal marker vimentin (green channel) and DAPI (blue channel).

As a key epithelial marker responsible for adherent junction, E-cadherin enables the cells to maintain epithelial phenotypes.^12^ 12h_5(NA)_ cells representing the lowest E-cadherin expression were selected to investigate the expression of mesenchymal proteins. Accordingly, to verify E-cadherin expression at the subcellular level, we took separated cellular fragments and analyzed them by western blotting. As shown in Figure 2A, 12h_5(NA)_ cells kept differentiating, and in the period of analysis, E-cadherin expression was totally lost in the membrane. Increased p-H3^(S10)^ expression in the nucleus proves their aggressive nature and increased proliferation. Depending on the suppression of E-cadherin, we examined N-cadherin expression as a mesenchymal marker. In the western blotting analysis, PC-3 (mesenchymal prostate cancer cell line) cells were chosen as a positive control, and 12h_5(NA)_ cells demonstrated increased N-cadherin expression in comparison to the control (Figure 2B). Further, when other 12 h CM-treated subpopulations were checked for mesenchymal markers, detectable levels of N-cadherin expression were found in each group (Figure 2C), suggesting that treatment of LNCaP cells with CM for 12 h suppresses E-cadherin and mediates N-cadherin expression. As the events occurring during EMT also include the increase of mesenchymal markers other than N-cadherin such as fibronectin and vimentin,^25^ the vimentin level was checked by immunofluorescence and its enhanced expression was detected in 12h_5(NA)_ cells (Figure 2D). Vimentin expression was also detected to be enhanced solely in the 6h_(NA)_ subpopulation (Figure S1A–C) without detectable levels of N-cadherin.

Consequently, the obtained results support that the inflammatory CM medium mediates EMT, and time of exposure to CM plays an important role in tumorigenesis, whereas 12 h CM-treated cells change their morphology toward EMT by expressing N-cadherin.

### Reduced NKX3.1 Expression Contributes to EMT

**Figure 3:**
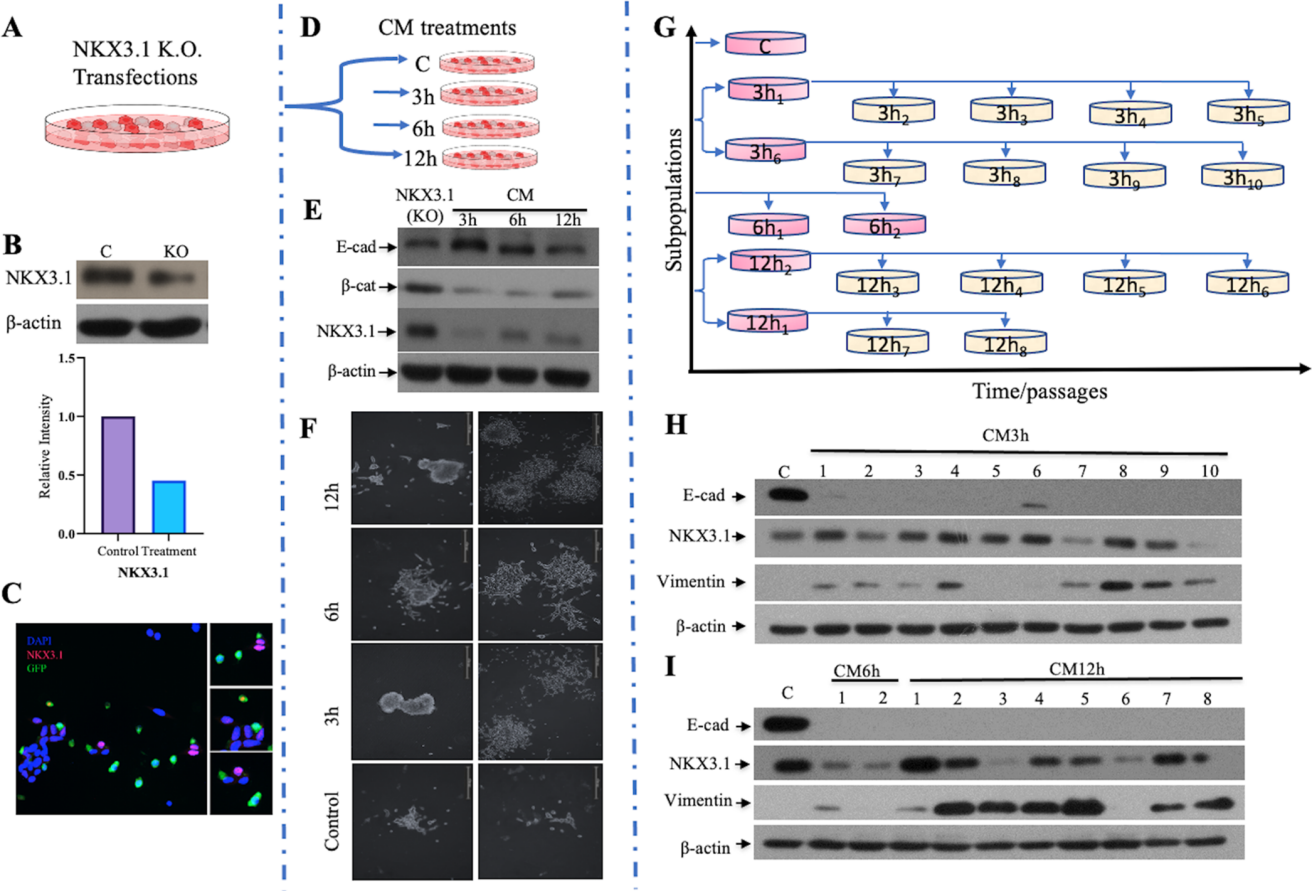
Alteration of EMT factors in NKX3.1-silenced cells. (A/D) Schematic diagram of inflammation-related migration assay in NKX3.1-silenced LNCaP cells and (G) obtained subpopulations. (B) Verification of NKX3.1 silencing by western blotting (up) and quantification of NKX3.1 expression compared to the control (down). (C) Immunofluorescence images of NKX3.1-silenced cells are shown in green GFP expression. Some cells still showed red NKX3.1 positivity. (E) Effect of CM in NKX3.1-silenced cells verified by western blotting. (F) Light microscopy images of migrated cells showing an aggressive phenotype compared to the control. Scale bars: 200 μm. (H/I) Control of E-cadherin, vimentin, and NKX3.1 expression in migrated subpopulations.

Previously, it has been shown that the inflammatory microenvironment suppresses NKX3.1 expression and causes LNCaP cell migration.^19^ However, although the loss of NKX3.1 provokes carcinogenesis in primary tumors, the cells maintain NKX3.1 expression even at a low level in metastatic prostate cancer. In order to represent the relatively low expression profile of NKX3.1 and to investigate its contribution to the EMT process in prostate cancer, we depleted the NKX3.1 expression by CRISPR (Figure 3A). NKX3.1 expression was detected to decrease to 45% based on western blotting results (Figure 3B), and the efficiency of silencing was measured as 31.4% based on immunofluorescence image analysis (Figure 3C).

Then, NKX3.1-silenced cells were propagated for the application of an in vitro inflammatory microenvironment model. Similar to a previous experiment, silenced cells were subjected to a migration assay following 3, 6, and 12 h of CM treatments (Figure 3D). Then, migrated cells were observed by light microscopy to demonstrate morphological changes. Consistent with our previous study,^19^ CM treatment resulted in NKX3.1 loss and reduced the expressions of adhesion molecules E-cadherin and β-catenin (Figure 3E). Subpopulations of NKX3.1(KO) and migrated cells showing distinct adhesion properties (Figure 3F) by the culture time were designated as shown in Figure 3G. Analysis of EMT-related protein expressions showed that the subpopulations of 3, 6, and 12 h of CM treatments resulted in distinct subpopulations with varying levels of vimentin as well as NKX3.1 expression in comparison to controls. Further, E-cadherin expression was detected to be lost in subpopulations except two with very low levels (1 and 6 of 3 h CM treatment) (Figure 3G/H). According to the obtained data, 3h_7_, 3h_8_, 6h_1_, and 12h_3_ subpopulations showing gained vimentin expression with varying levels of NKX3.1 losses were chosen for further investigation.

### NKX3.1 Expression is Negatively Correlated to In Vitro Colony Formation Ability

**Figure 4:**
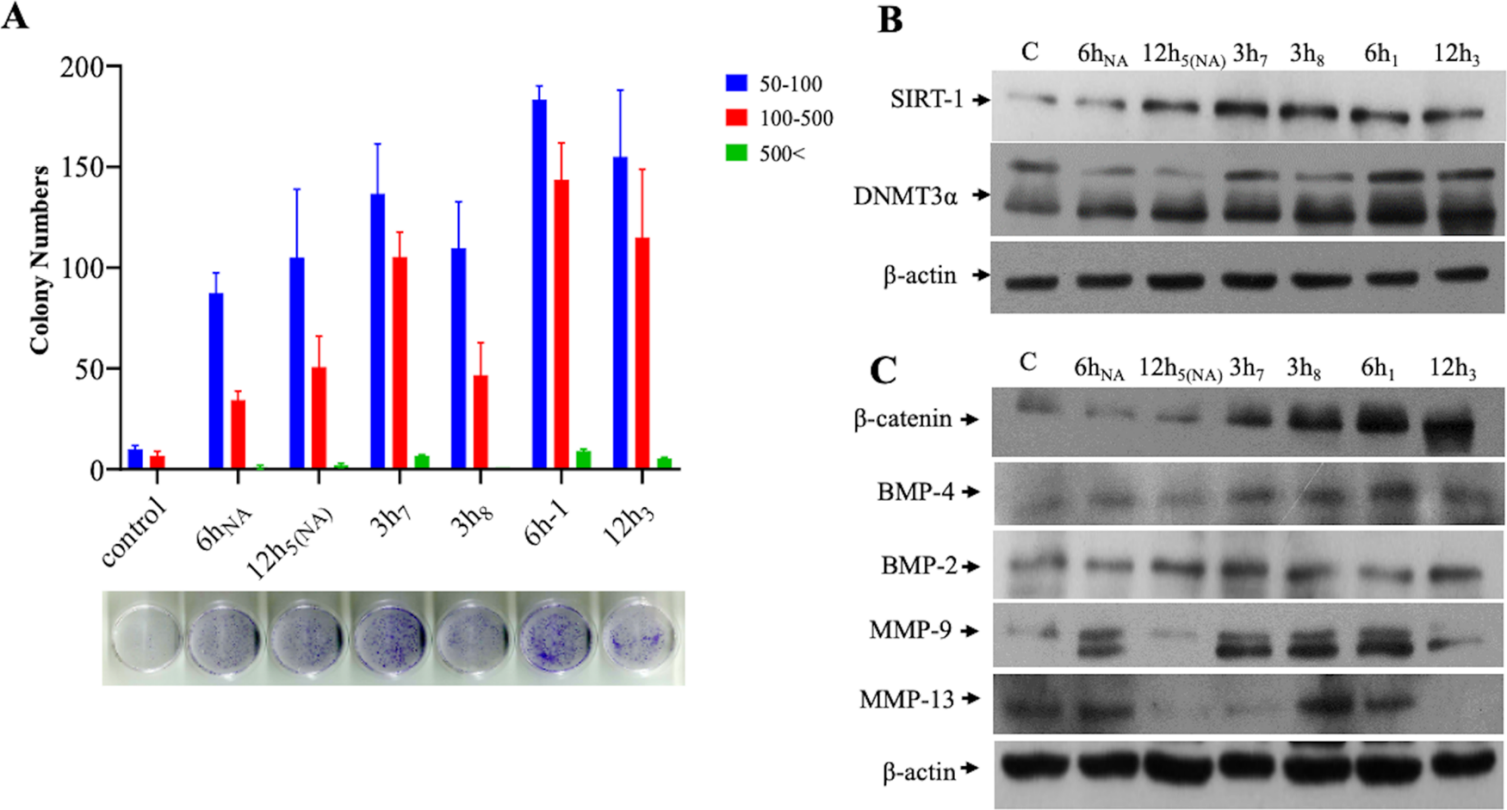
Investigation of the heterogeneous phenotype of subpopulations. (A) Colony-forming abilities of selected subpopulations. The graphical representation of the number of colonies at different sizes is shown. Each color represents a different size [pixel] of counted colonies. Analysis was performed using Image J software, and data are means ± SD of three independent experiments (*p* < 0.001). (B) Epigenetic changes investigated by SIRT and DNMT3a. (C) Expressions of metastasis-related BMP and MMP gene family members in subpopulations.

To distinguish the NKX3.1 loss among CM-mediated migration effects, migrated subpopulations were examined for colony formations. Results showed that NKX3.1-silenced subpopulations form larger colonies with a higher number for each size in comparison to the control. Colony numbers were also determined to be correlated to the NKX3.1 expression level as subpopulations 3h_8_ (Figure 4A) and 6h_NA_ and 12h_5(NA)_ (Figure S1A), which have relatively high NKX3.1 expression formed fewer colonies. In addition to this, the NKX3.1-silenced subpopulations exhibited higher migration capacity in comparison to control cells migrated upon the effects of inflammatory microenvironment solely (Figure 4A). These results suggested that the increased vimentin expression in varying levels in combination with decreased NKX3.1 expression could be an important factor augmenting aggressive tumor formation.

### Investigation of Heterogeneous Phenotype by Epigenetic and Metastatic Protein Alterations

Epigenetic modification is one of the major contributors to altered gene expression in cancer development. However, the most commonly observed epigenetic changes are aberrant DNA methylation, histone acetylation, and deacetylations. SIRT1 and DNMT3a are essential classes of modifiers in epigenetics.^26,27^ Nakane K. and coauthors showed that SIRT1 promotes EMT in PCa cells. Likewise, SIRT1 is enrolled via ZEB1 to bind the E-cadherin proximal promoter; SIRT1 deacetylates the histone H3 and inhibits the RNA polymerase II binding, resulting in the transcription blockage of E-cadherin. Thus, SIRT1 acts as a positive regulator of EMT to influence the metastatic growth of prostate cancer cells.^28^ Accordingly, the investigation functional role of DNMTs in PCa progression reveals DNMT3a to be upregulated in high Gleason score tumors.^27^

In order to find out the contribution of these epigenetic regulators to inflammation-induced migration in prostate cancer, our migrated subpopulations were examined. Although, there were slight variations in expressions between subpopulations, overall SIRT1 and DNMT3a expressions in NKX3.1-silenced subpopulations were relatively higher than in the control, suggesting that epigenetics is also subject to change in these phenotypes (Figure 4B).

One of the relevant proteins, which play an important role in prostate cancer bone metastasis is BMP proteins, a member of the TGFβ superfamily.^29^ BMPs are known to be involved in metastatic progression and tumorigenesis of many types of cancers. Most investigated family member BMP-4 enhances epithelial–mesenchymal transition (EMT) and stem cell properties in both mammary epithelial and breast carcinoma cell lines. In addition, BMP-4 upregulates the colony-formation efficiency,^30^ but functional studies have revealed contradictory roles of BMP-2 in both cancer promotion and inhibition. Although previous data showed that BMP-2 inhibited cancer cell growth in vitro and in vivo by inducing G1 arrest and apoptosis,^31^ the latter study demonstrates that BMP-2 is a driving factor for promoting EMT and breast cancer stemness.^32^ In our case, β-catenin, BMP-4, and MMP-9 expressions were significantly high in NKX3.1-silenced cells. While a slight increase in BMP-2 was also detected in all subpopulations, higher expression of MMP-13 was found in two of the NKX3.1-silenced subpopulations (Figure 4C).

### Coexpression of E-Cadherin and Vimentin in Inflammation-Related Migratory LNCaP Subpopulations

As the 6h_NA_ subpopulation changed its morphology to a monolayer and grew enough to make some analysis, we used them to explore the similarity of 6h_NA_ with other low-adhesive migrated cells. Whereas, dissimilar to 12h_5(NA)_ cells, 6h_NA_ cells did not show any N-cadherin expression, and the E-cadherin level was close to the control (Figure S1A). Afterward, when vimentin expression was checked by western blotting, opposite results were obtained compared to the previous N-cadherin data, where 6h_NA_ cells showed high vimentin expression (Figure S1B). Previously, it has been shown in breast cancer samples that the coexpression of E-cadherin and vimentin is associated with the most aggressive phenotype and poor prognosis; hence, increased E-cadherin expression may not always play roles in tumor suppression.^33^ Immunocytochemistry analysis proves the validity of vimentin expression in subpopulations (Figure S1C). Simultaneously, the data suggest that the 12h_5(NA)_ cells had a reversible-transitional EMT process, in which some of the cells may revert to previous expression patterns “at certain levels” despite cellular progression when inflammatory exposure is discontinued. In humans, NKX3.1 downregulation is acknowledged as one of the earliest events in prostate cancer initiation.^34^ Upon androgen deprivation, NKX3.1 expression is rapidly lost, while it is quickly restored after androgen readministration for prostate maintenance.^35^ Previously, the loss of functional NKX3.1 has also been demonstrated in our laboratory under inflammatory conditions in vitro.^17^ Interestingly, we have observed that the migration-selected subpopulations increased NKX3.1 expression at longer periods of propagation (Figure S1B), indicating that the NKX3.1 level might be kept high with some other factors than CM itself. Since the NKX3.1 expression is correlated to AR transactivation, we investigated the nuclear translocation of AR using immunofluorescence labeling for our subpopulation of interest (Figure S1C). Thereafter, we studied AR transactivation using reporter assay by administering the ARE sites into cells ectopically. Taken together, AR transactivation was found to be higher in the 12h_5(NA)_ subpopulation in comparison to control LNCaP cells with or without the administration of synthetic androgen R1881, though the AR translocation levels did not change much (Figure S1D).

Overall, the data show that inflammatory microenvironment-related changes in migrating cells are increased by altered NKX3.1 expression, where NKX3.1 is an important switch regulating the cellular response through androgen-dependent mechanisms in both directions. In the case of higher vimentin expression, NKX3.1 loss increased the epithelial transition to a mesenchymal phenotype, which is clearly associated with a higher growth rate in our derived subpopulations. Thus, our data imply that NKX3.1 is an essential and sole factor for regulating the cellular progression and colony formation in androgen-responsive cells in prostate cancer.

### Proteomic Profiling Identifies Differentially Regulated Proteins during Migration

**Figure 5:**
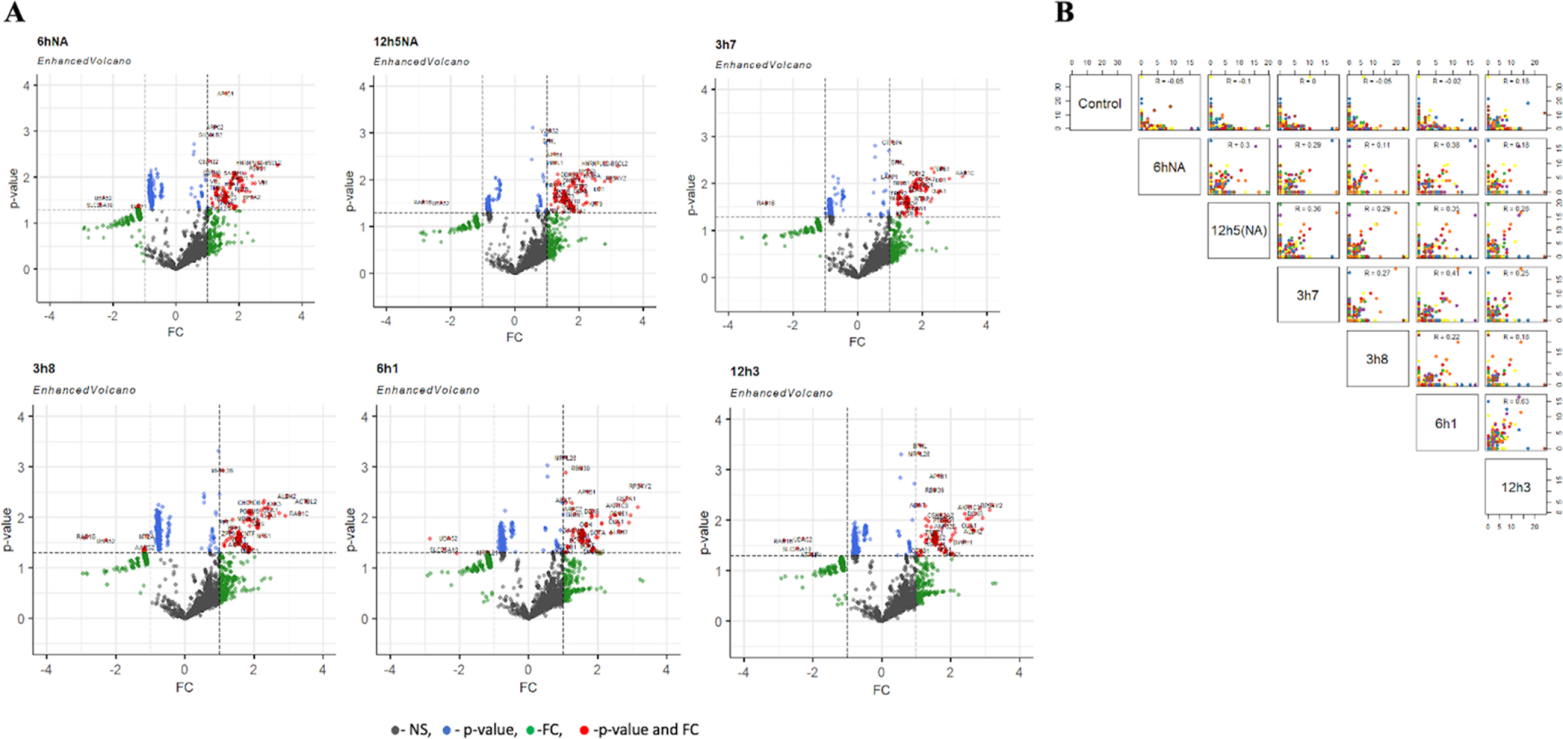
(A) Differentially expressed proteins in each subpopulation identified by the volcano plot. Genes that are nonspecific are marked black, only *p*-value-specific are marked blue, only FC-specific are marked green, and those that are significant according to the p-value and the FC threshold are marked red. (B) Biological replicate data plotted as a scatter plot matrix; the difference between subpopulations is represented by the Pearson correlation coefficient (*R*).

To identify essential programs associated with inflammatory CM-mediated cell migration, advantages of quantitative proteomics were taken for the migrated subpopulations. To determine the measurement error, two technical replicates were performed for each subpopulation in systemic quantitative profiling of inflammation-mediated cell migration. All raw MS data were processed using Proteome Discover software. In total, 4715 proteins were identified. The number of determined proteins for each cell group is represented in the table (Table S1). Further data analysis has been done using the R programming language on the R studio platform. First, each technical replicate was compared using Pearson correlation to verify the correction of the experimental workflow. As shown in Supporting Figure 2 (Figure S2), the ratio for each replicate was above 0.96 (*p* < 2.2 × 10^–16^), indicating the reproducibility of the presented workflow. To highlight the most significant changes, the p-values and fold changes were measured on Microsoft Excel and plotted to the volcano plot in R studio. Correspondingly, multivariate data of samples with a total of 985 proteins were found to be significantly expressed according to the *p*-value ≤ 0,05 cutoff. In the volcano plot graph, the fold change is represented as log 2, and the p-value is represented as log 10. The fold change threshold (FC) was chosen to be 1; for the *p*-value, this number corresponds to 0.05 (Figure 5A). Genes with decreased expression are located on the left side of the *x*-axis, while the genes of which expression is increased are illustrated to the right. Genes with statistically significant differential expression lie above a horizontal threshold and are marked in red. Further, to elucidate the similarities and differences between biological repeats, all subpopulations with control cells were compared against each other by Pearson correlation. Positive and negative relationships are represented in Figure 5B. Control cells were negatively correlated with four subpopulations except 12h_3_, with *R* = 0.18. 3h_7_ cells have no correlation with the control and are represented as *R* = 0. Subpopulations are positively correlated with each other at different R levels (Figure 5B).

**Figure 6:**
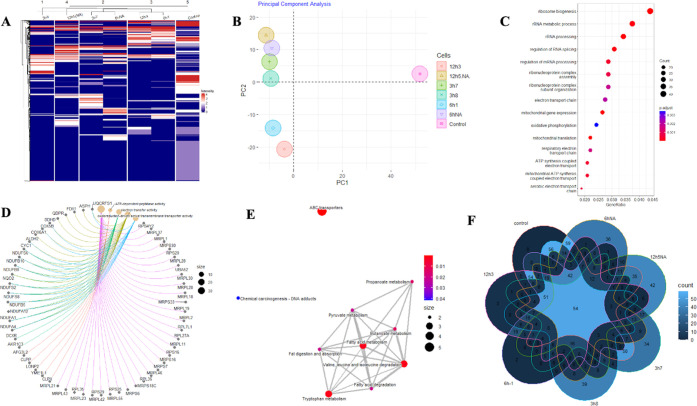
Differential protein analysis and GO enrichment analysis of the quantitative proteome. (A) Nonsupervised hierarchical clustering of the top 985 most variant proteins. (B) Principal component analysis (PCA). (C) Gene ontology (GO) term enrichment analysis of differentially expressed proteins. (D) GO results by Cnetplot. (E) KEGG pathway enrichment analysis. (F) Venn diagram showing the distribution of the 985 identified proteins in each subpopulation.

Heatmaps of all proteins selected are shown in Figure 6A. The dendrogram of the heatmap was created by unsupervised *k*-means clustering, meaning that the hierarchical clusters formed were based purely on the similarities and dissimilarities among the samples by the expressions of the proteins. According to hierarchical clustering, the control group has less association with the migrated subpopulations. “3h_7_” and “6h_NA_”, as well as “12h_3_” and “6h_1_” subpopulations created small units under the “12h_5(NA)_” cluster. “3h_8_” cells are presented as a separate cluster.

Next, the presented data were evaluated using principal component analysis, and three clusters corresponding to different phenotypes were observed (Figure 6B), reflecting a close correlation between morphology and protein expression levels. All migrated subpopulations differed from the control. The NKX3.1-silenced clusters 12h_3_ and 6h_1_ set close to each other and the remaining 12h_5(NA)_, 6h_NA_, 3h_7_, and 3h_8_ formed the last group located far from the control.

To associate the subpopulation phenotype with a group of proteins and to obtain a broad perception of protein expression differences, a gene set enrichment analysis was performed via the “ClusterProfiler” package in R. Accordingly, mitochondrial, ribosomal, and metabolic processes were the most significantly enriched biological processes; mitochondrial complex and ribosomal subunits were the most significantly enriched cellular components; and ATP-dependent peptidase, ligase, and NAD(P)H dehydrogenase activity were the significantly enriched molecular functions (Figure S3). The top 15 GO (gene ontology) terms in which the differentially expressed proteins were enriched according to the gene count are presented in Figure 6C. The Cnetplot of GO results were combined to show the potential biological complexity of all chosen proteins that may belong to multiple annotation categories. Most of the proteins were overlapped in the “electron transfer activity”, “oxidoreductase activity, acting on NAD(P)H, quinone or similar compound as acceptor”, and “oxidoreduction-driven active transmembrane transporter activity” (Figure 6D). Next, KEGG pathway enrichment analysis was done on these 985 proteins (Figure 6E), and the results showed that the most significant pathway for chosen protein enrichment was “ABC transporters.”

However, several studies have demonstrated that ABC transporters play an additional fundamental role in tumor biology; there is strong evidence that these proteins might be involved in the transport of tumor-augmenting molecules and/or protein–protein interactions that affect cancer aggressiveness, progression, and the patient prognosis. Apart from these findings, the relationship between cancer-causing inflammation and ABC proteins is almost intuitive due to their efflux function, since inflammation is regulated by multiple molecules, some of which are substrates of these molecular pumps. In addition, there were different degrees of enrichment in some amino acid metabolisms, fatty acid metabolisms, and “chemical carcinogenesis-DNA adducts.” Taken together, unique quantifiable proteins of each subpopulation in comparison to the control group, which were shown by the Venn diagram (Figure 6F), resulted in 15 proteins common in expression in all migrating subpopulations (Table S2).

## Discussion

Depending on where the solid tumors develop, various etiological factors might influence the pathophysiology and the progression of the cancer, which then emerges in time as a serious disease. One of these is the well-known tumor microenvironment, which might trigger tumor surveillance mechanisms upon activated immune cell infiltration. Hence, inflammation is a defense mechanism that produces the secretion of proapoptotic cytokines; it might augment the cancer progression at certain stages of tumor growth. Thus, the mechanism of switching slow-growing tumors into fast-growing ones is still under investigation in many studies.

In our study, we investigated inflammatory microenvironment-mediated prostate cancer progression in LNCaP and NKX3.1-silenced LNCaP cells by using a CM medium as a source of inflammation. Our results revealed that a period of exposure to the CM medium defines changes occurring in the cell. LNCaP cells have epithelial behavior by expressing E-cadherin, and as our results verified, CM exposure changed their epithelial morphology to mesenchymal. These changes were verified by various methodologies including the analysis of expressions of N-cadherin and vimentin in subpopulations. It is commonly acknowledged that the epithelial marker E-cadherin loss and the mesenchymal marker N-cadherin gain imply that the EMT and mesenchymal–epithelial transition (MET) is a transitional process with two distinct cell populations, epithelial and mesenchymal. However, recent studies revealed that there are numerous variations with specified gene expression patterns of the mesenchymal marker vimentin between these two states. Here and in previous studies, it was shown that the EMT appears in a gradual manner and is characterized by several cellular stages expressing different levels of EMT markers, specifically demonstrating intermediate morphological, transcriptional, and epigenetic features in both types of cellular states. As shown in our results, a gain of N-cadherin and vimentin expression and a loss of E-cadherin were observed at the beginning of growth. After subpopulations were kept in culture, the regain of E-cadherin expression in different levels by keeping vimentin expression was demonstrated. Since the factors enforcing the states shifting to each other are not well defined, the position of cells involved could be referred to as partial, incomplete, or hybrid EMT states, as suggested.^36^ Cells in this hybrid phenotype have mixed epithelial and mesenchymal properties such as decreased adhesion and increased migration, respectively, thus allowing them to form clusters at a particular stage of EMT. These clustered clones coexpress both epithelial and mesenchymal markers at varying levels.^37^ Our results show that an inflammatory CM medium can cause a resemble hybrid phenotype. The subpopulations also showed a higher growth rate in comparison to parent LNCaP cells, which was studied with the p-H3^S10^ level.

Although the underlying cellular processes are not well understood, inflammation has long been linked to the loss of key tumor suppressor gene NKX3.1 and prostate cancer progression.^13^ However, there have been conflicting reports regarding the NKX3.1 expression with tumor progression. In the study of metastatic carcinoma of an unknown primary site, Gurel et al. found that the vast majority of metastatic prostate adenocarcinomas (98.6%) retained NKX3.1 expression, while only 1 of 383 cases of nonprostatic tumors expressed NKX3.1.^38^ Another similar work was provided by Huang et al., demonstrating that PSMA and NKX3.1 are more sensitive markers than PSA for bone metastasis of PCa, following decalcification.^39^ Based on the available data, we developed a similar phenotype by suppressing the expression of NKX3.1 at a certain level. The resulting cells were used for CM-mediated cell migration. In contrast to the previous experiment with unaltered LNCaP cells, subsets of LNCaP cells with suppressed NKX3.1 expression readily adhered to the plate and began to proliferate, and subsets were obtained from each CM-treated cell. The clusters created by the migrated cells were collected separately and kept in an incubator for further characterization. Interestingly, a very short period of cytokine exposure was quite effective for these cells and changed cellular morphology within a few hours, thus maintaining their transient phenotype for a longer period of propagation. EMT-related outcomes of the NKX3.1 loss in the inflammatory microenvironment are suggested to be strictly dependent on dose and time of exposure because of various cellular functions of NKX3.1 in the regulation of oxidative stress and DNA damage recognition, which directly affect cell survival.

Later, expression levels of a wide range of proteins involved in EMT-related pathways were investigated in all subpopulations, suggesting that different combinations of different levels of EMT-regulating factors enable different phenotypes. Changes in EMT markers and enhanced migration capacity upon suppression of NKX3.1 also raise the question of the role of NKX3.1 in adhesion properties and survival capacity of low-adherent cells, which may lead them to develop anoikis resistance during migration. Signaling pathways regulating these cellular processes are suggested to need further investigation under NKX3.1 suppressed conditions in future studies. While SIRT1 and DNMT3a alterations occurred in all CM-treated subpopulations, β-catenin, BMP-4, and MMP-9 increases were observed especially in NKX3.1-silenced subpopulations. Thus, increased SIRT1 led transformed cells to better tolerate oxidative stress coming from their high proliferation rate,^40^ and increased DNMT3a, as a methyltransferase, resulted in an aberrant expression of many EMT-related factors.^41^ However, a few of these subpopulations showed more aggressive phenotypes to form larger cell clusters in colony-formation assay demonstrating enhanced β-catenin, BMP-4, and MMP-9 expressions with the NKX3.1 loss, suggesting that the induction of these EMT-regulating factors might occur in combination with the former changes to complement the relatively more invasive phenotype of subpopulations. Since BMP-4 and MMP-9 contribute to extracellular matrix remodeling, which is a major component of tumor progression, an increased expression of these proteins likely facilitates abnormal ECM-promoting cell migration in NKX3.1-silenced cells.

All subpopulations were subjected to proteomic analysis. A nano-MS/MS-based quantitative proteomics approach was used to study the inflammatory microenvironment-mediated migration of LNCaP cells and its various developmental subpopulations. In total, 4715 proteins were identified and 985 of them were found to be significantly expressed according to the *p* ≤ 0.05 threshold. Except for *p*-value measurement, which is performed using the Microsoft Excel “Real Statistics” plug-in, the rest of the bioinformatics analysis of differentially expressed proteins was performed in R programming. The results revealed that the duration of exposure to the inflammatory microenvironment and NKX3.1 expression played a major role in establishing subpopulations. Notably, 12h_3_ and 6h_1_ cells with longer CM exposure as well as NKX3.1 suppression were more affected and formed discrete clusters, as shown in PCA and hierarchical heatmap analysis. Recent discoveries point to a broader role for the dysregulation of ribosome biogenesis in the development and progression of most spontaneous cancers.^42^ Notably, our GO analysis results showed that “ribosomal biogenesis” was most significantly enriched in differentially expressed proteins. RNA metabolic processes and mitochondrial processes were also noted in the GO enrichment assay. It is well known that cancer cells reprogram metabolic processes to support tumor cell proliferation and survival requirements, and mitochondria are a major part of supporting tumor cell metabolism. The presented results of the GO analysis were confirmed by the KEGG pathway enrichment analysis, where the pathways associated with “ABC transporters” were the most significant. As known from the literature, ATP-binding cassette (ABC) transporters are a family of transporter proteins responsible for drug resistance and low bioavailability of drugs by pumping various drugs out of cells due to ATP hydrolysis. Also, they have an important role in cancer aggressiveness.^43^ Gaining expression of such proteins in subpopulations may serve as further evidence of the role of the inflammatory microenvironment in the development of cancer. Other pathways identified in the KEGG pathway analysis were mostly metabolism-related pathways, together with “Chemical carcinogenesis-DNA adducts.”

## Conclusions

There are many reports in the literature explaining the role of NKX3.1 at varying levels in EMT during prostate cancer development and metastasis. In this study, we aimed to reveal the relationship of NKX3.1 with inflammation-related prostate cell migration. As a result, we have explained that NKX3.1 is re-expressed in inflammatory migrating cells positively affecting cancer development. However, NKX3.1-silenced and migrated clusters had more aggressive phenotypes than NKX3.1-expressing ones, implying that the loss of NKX3.1 leads prostate cells to proceed to advanced stages of EMT. Further, vimentin and NKX3.1 expressions are favored in prostate cancer cell line LNCaP during EMT, where genetic changes associated with inflammation as a dynamic process required for tumor progression. Comprehensive proteomic analysis in LNCaP cells revealed similarities and differences between subpopulations according to NKX3.1 and CM-exposure time points. Analysis of differentially expressed genes showed altered ribosomal and metabolic processes, as predicted by many studies; this requires more studies. ABC transporters were found to be important under the inflammatory environment, and the loss of NKX3.1 also needs further investigation in the future.

Since, EMT has been implicated in carcinogenesis and confers metastatic properties upon cancer cells by enhancing mobility, invasion, and resistance to apoptotic stimuli, TNFα-rich CM may be responsible for changes in epithelial–mesenchymal morphology; chronic exposures to an inflammatory environment might be the major cause of a more aggressive EMT hybrid phenotype. Future studies are required to conclude.

Overall, the data suggest that NKX3.1 is an important regulator of carcinogenesis and is required for EMT phenotype regulation, perhaps under hormone influence. The transitional phases of EMT–MET could be more certain at least for the prostate cancer cell lines. A better understanding of the major players of inflammation in a tumor microenvironment still needs more extensive protein/expression profiling studies, especially in in vivo settings.

## Supporting Information Available

High NKX3.1 expression together with AR transactivation, where the subpopulations exhibited variable vimentin/N-cadherin expressions (Figure S1); consistency between technical replicates of each cell group by scatter plots (Figure S2); gene ontology (GO) term enrichment analysis (Figure S3); protein counts detected in samples (TR-technical replicates) (Table S1); and proteins coexpressed in migrating subpopulations gathered from Venn diagrams (Table S2) (PDF)
Raw data from proteomics analysis was added as an Excel (XLS) spreadsheet (XLSX)

## Abbreviations

**AR**: androgen receptor
**PCa**: prostate cancer
**EMT**: epithelial–mesenchymal transition
**MET**: mesenchymal–epithelial transition
**NKX3.1**: neurokinin receptor 3 homeobox 1
**TNFα**: tumor necrosis factor-α
**TGFβ**: transforming growth factor-β
**AKT**: RAC-α serine/threonine-protein kinase
**LPS**: lipopolysaccharides
**PMA**: phorbol 12-myristate 13-acetate
**ABC**: ATP-binding cassette
**KEGG**: Kyoto encyclopedia of genes and genomes
**CM**: conditioned medium
**PD**: proteome discoverer
