# Supplementary material for: NKX3.1 Expression Contributes to Epithelial–Mesenchymal Transition of Prostate Cancer Cells
Source: ACS Omega. 2023 Sep 1;8(36):32580–92. doi: 10.1021/acsomega.3c03127 (PMC10500679; doi:10.1021/acsomega.3c03127)
Supplement: Supplementary file 1 — ao3c03127_si_001.pdf [file ao3c03127_si_001.pdf]

Supplementary Information

NKX3.1 Expression Contributes to Epithelial-Mesenchymal Transition of Prostate Cancer Cells

Author List

Iroda Saydullaeva [1,3], Bilge Debelec Butuner [2], Kemal Sami KORKMAZ [1*]

[*]Corresponding authors

Institution Identification

[1]Ege University, Faculty of Engineering, Department of Bioengineering, Cancer Biology Laboratory, Izmir, Turkey

[2]Ege University, Faculty of Pharmacy, Department of Pharmaceutical Biotechnology, Izmir, Turkey

[3]Present address: IYTE Gulbahce Campus, Fac. of Science, Gulbahce, Izmir, Turkey, 35040.

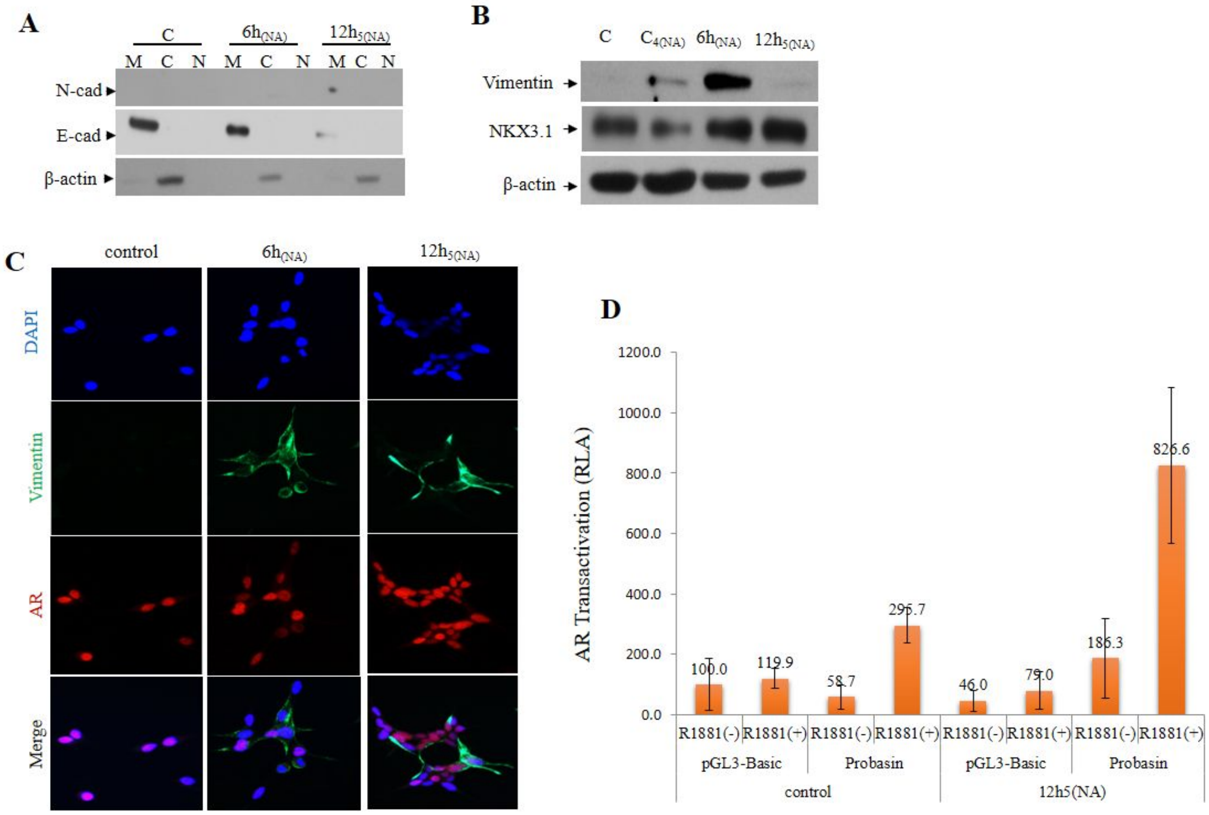

Figure S1. High NKX3.1 expression together with AR transactivation. A) Verification of mesenchymal N-cadherin and epithelial E-cadherin expressions in 6h $_{(NA)}$ cells and12h$_{5(NA)}$ cells B) The subpopulations exhibited variable vimentin expression together with migrated controls, where NKX3.1 expression was consistently higher in CM-treated subpopulations. C) Both AR expression, nuclear translocation, and vimentin expressions are seen in immunofluorescence microscopy images, where 12h$_{5(NA)}$ showed higher vimentin expression than in westerns. DAPI was used for nuclear staining. D) AR responsive rat probasin promoter was used and showed that 12h$_{5(NA)}$ had increased AR transactivation, putatively augments the NKX3.1 expression compared to control cells.

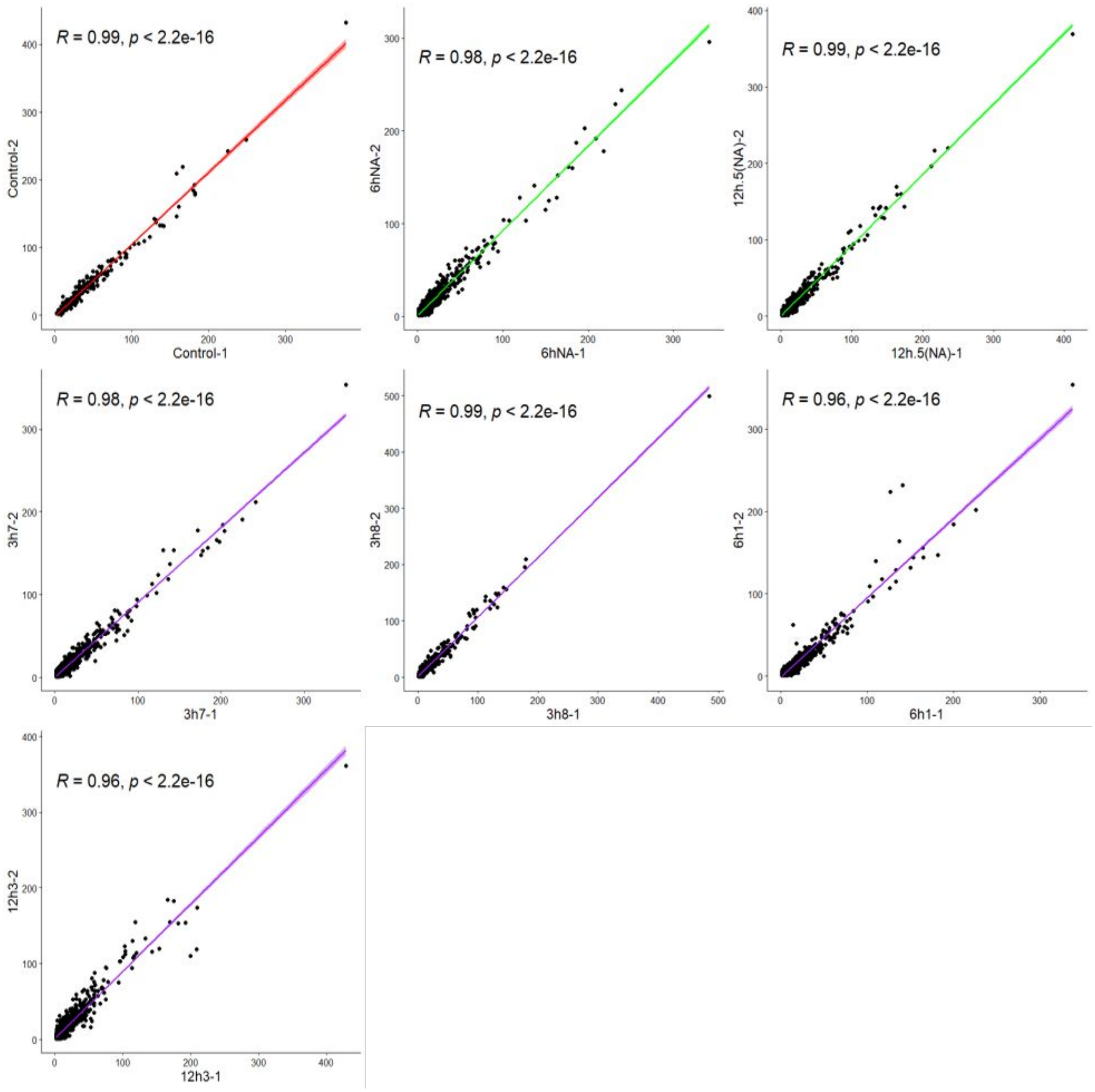

Figure S2. Consistency between technical replicates. Scatter plots show the correlation between the two replicates for each cell group. R indicates the Pearson correlation coefficient.

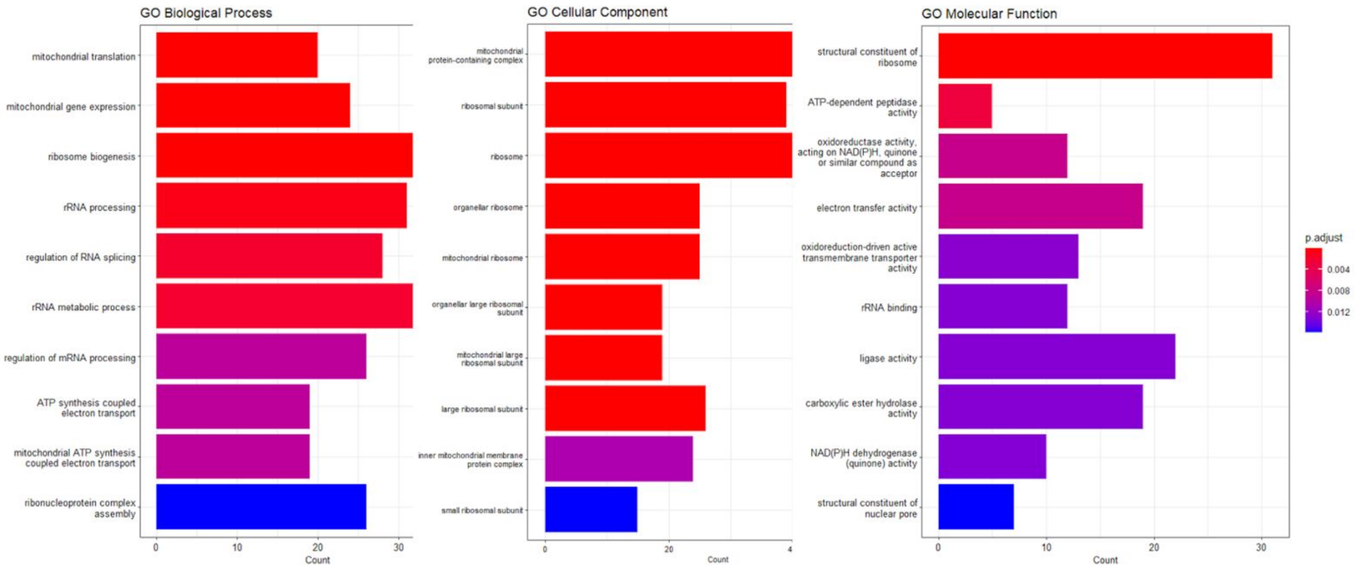

Figure S3. Gene Ontology (GO) term enrichment analysis. GO terms of the categories of Biological Processes, Cellular Components, and Molecular Functions are presented separately.

Table S1. Protein counts detected in samples (TR-technical replicates).

**Cell Type**	**TR-1**	**TR-2**	**number of elements**
Control	286	2084	2088
$6h_{NA}$	1863	1957	2382
$12h_{5(NA)}$	1934	2022	2442
$3h_7$	1949	2053	2509
$3h_8$	1868	2102	2478
$6h_1$	2002	1721	2337
$12h_3$	1995	1998	2585

Table S2. Proteins co-expressed in migrating subpopulations are gathered from Venn diagrams and listed the best common hits in comparison to controls.

Protein ID	Gene Name
Q92667	A-kinase anchoring protein 1(AKAP1)
H3BQZ7	HNRNPUL2-BSCL2 readthrough (NMD candidate)(HNRNPUL2-BSCL2)
P61916	NPC intracellular cholesterol transporter 2(NPC2)
Q9NTK5	Obg like ATPase 1(OLA1)
Q9NXG2	THUMP domain containing 1(THUMPD1)
P05091	aldehyde dehydrogenase 2 family member(ALDH2)
O60610	diaphanous related formin 1(DIAPH1)
Q7Z4W1	dicarbonyl and L-xylulose reductase(DCXR)
Q9NV88	integrator complex subunit 9(INTS9)
O00505	karyopherin subunit alpha 3(KPNA3)
Q9BRJ2	mitochondrial ribosomal protein L45(MRPL45)
Q6L8Q7	phosphodiesterase 12(PDE12)
Q06323	proteasome activator subunit 1(PSME1)
O43765	small glutamine rich tetratricopeptide repeat co-chaperone alpha(SGTA)
Q12874	splicing factor 3a subunit 3(SF3A3)
